# Bacterial melanin crosses the blood–brain barrier in rat experimental model

**DOI:** 10.1186/2045-8118-11-20

**Published:** 2014-08-25

**Authors:** Tigran Petrosyan, Anichka Hovsepyan

**Affiliations:** 1 Armenian State Institute of Physical Education, 11 Alex Manukian, Yerevan 0028, Armenia; 2 SPC “Armbiotechnology” NAS RA, 14 Gyurjyan Str., Yerevan, Armenia

**Keywords:** Bacterial melanin, Blood–brain-barrier, Transport, Labeling

## Abstract

**Background:**

Bacterial melanin has been proven to stimulate regeneration after CNS lesions. The purpose of this study was to test, whether bacterial melanin can enter the brain via the blood–brain barrier (BBB).

**Methods:**

Bacterial melanin (BM) was radioactively labeled by the iodobead method and used to test the BBB permeability after systemic injection into rats. The unidirectional influx rate from the blood was calculated by multiple-time regression analysis. A subgroup of the animals was co-injected with non-labeled BM to determine if BM has a saturable transport across the BBB. The levels of radioactivity were determined in the serum and tissues. Arterial blood was sampled to obtain the level of I-BM at different time points after injection. After systemic perfusion with saline, animals were decapitated and brain, spinal cord, liver and kidney samples were obtained and homogenized to test the I-BM level.

**Results:**

Study results showed that radioactively-labeled bacterial melanin crossed the BBB, was enzymatically stable in blood and in brain parenchyma. Entry to brain was reduced when non-labeled BM was also present. Circulating melanin entered all regions of the CNS but the uptake was higher in lumbar spinal cord, thalamus, hypothalamus and substantia nigra. Liver and kidneys had high uptake rates of BM.

**Conclusions:**

These results show that bacterial melanin has saturable transport across the BBB and selectively targets some CNS regions. Such transport may contribute to the neuroprotective action of bacterial melanin.

## Background

Peptide and protein drugs have shown great potential for the treatment of various neurodegenerative diseases. A major challenge in the pharmacokinetics of such drugs is the delivery of peptides or proteins across the blood–brain barrier (BBB). Despite the importance of the BBB for neurotherapeutics, the BBB receives insufficient attention in research. Both, the lack of effort in developing solutions to the BBB problem, and the minimal BBB transport of the majority of all potential neuroprotective agents, has led consequently to the present situation in neurotherapeutics. As a result there are few effective treatments for the majority of neurodegenerative disorders. This situation can be changed by an accelerated effort to create a knowledge base in the fundamental transport properties of the BBB, and the molecular and cellular biology of the brain capillary endothelium [1]. There are many methods to quantify the BBB permeability to various test compounds [2]. Different *in vivo* approaches include pharmacokinetic analysis, intracerebral microdialysis, positron emission tomography, magnetic resonance imaging, and histochemistry. *In vitro* techniques typically use tissue culture models of cerebrovascular endothelial cells either alone or co-cultured with astroglia. *In vivo* techniques to test BBB permeability include *in situ* brain perfusion, the intravenous injection/multiple time point approach, and the brain efflux index procedure. These techniques can be used for a broad array of substances, ranging from synthesized new chemical entities to biotechnology-derived peptides and proteins [3].

For the last decade the efforts of researchers to investigate neurodegenerative disorders have been focused on the problem of regulating activity in dopaminergic neurons [4]. The goal has been not only to identify the underlying mechanisms of altered activity in such neurons, but also the routes for regulation and methods used to prevent changes in neuronal activity in such patients. Treatment options for neurodegenerative disorders have provided clinicians with a number of neuroprotective agents, each with a different chemical composition and mechanism of action. Many of the offered options aim to support cell survival and accelerate posttraumatic recovery of CNS function.

A number of studies have shown the neuroprotective action of melanocyte-stimulating-hormone on locomotor recovery following CNS lesions [5-7]. Currently melanins of various origins are being actively studied and applied as therapeutic agents. Melanins are multicoloured pigments of polymer structure. They are unique transmitters of energy with the property of an amorphous semiconductor. They can absorb the energy and convert it into various types of energy [8-10]. Melanins break free radical chain reactions and accomplish antioxidant protection. These unique abilities of melanin explain its presence in tissues and organs connected with impulse transmission, such as skin, retina, inner ear and nervous system. Disorders of melanin metabolism may be involved in the etiology of such diseases as Parkinsonism, senile macular degeneration, and senile deafness [11,12]. This pigment is also relevant to the well-known association between pigment abnormalities and deafness (Warrensburg’s and Usher’s syndromes) [13,14]. Furthermore, Alzheimer’s disease and Down’s syndrome were observed to be also accompanied with pathological disorders in melanin metabolism [15]. The majority of synthetic and natural melanins are insoluble in water. This significantly complicates preparation of melanin-containing pharmacological and cosmetic preparations. Obtaining low-cost soluble natural melanin can essentially stimulate and speed up the use of melanin in medicine, cosmetology and other fields. For the first time, a melanin-synthesizing bacterial strain, *Bacillus thuringiensis,* with a high level of pigment synthesis was obtained. The ecologically-safe technology of biosynthesis, isolation and purification of bacterial melanin (BM) has been elaborated [16]. Biotechnologically-obtained bacterial melanin exhibits a similar infra-red absorption spectrum to synthetic melanin and contains quinolic and phenolic substances and around 20% amino acids after acid hydrolysis. The molecular weight of the purified melanin determined by SDS-PAGE was 4 kDa and the electromagnetic spin resonance spectrum of the purified microbial melanin was a slightly asymmetric singlet without hyperfine structure with about 7 Gauss width of the line between points of the maximum incline and g = 2.006. The concentration of paramagnetic centers in melanin is 0.21 · 1018 spin/g. BM is homogenous in polymeric structure and does not contain polymers of tyrosine of different length [16].

The biological activity of melanin has been studied on both animals and plants [17-19]. In experiments on laboratory animals (Wistar rats) with brain surgical trauma, it was revealed that BM facilitated the recovery of instrumental conditioned reflexes after unilateral ablation of sensorimotor cortex that had caused paresis of limbs [20]. In addition, low doses of BM accelerated the recovery of physiological functions lost because of nervous tissue damage [21]. In a previous experimental series, we have shown the neuroprotective action of BM after neurotrauma in the CNS in that BM accelerates motor recovery and enhances regeneration after substantia nigra lesions [22]. Injection of BM increases electrical activity of substantia nigra dopaminergic neurons [23], although it remained unclear whether the BM is able to cross the BBB or not. The goal of the present study was to test the ability of BM to cross the BBB using the method of radioactive labelling.

## Methods

### Animals

Experimentally-naïve male Wistar rats (n = 12) were used at 3–6 months of age and weighed 180–200 g at the start of experiment. Rats were housed with their littermates in plastic boxes covered by a wire lid and were maintained on a standard light–dark cycle with food and water available *ad libitum*. Animals were maintained and handled in accordance with institutional guidelines and national and international laws and policies (EEC Council Directive 86/609, OJ L 358, 1, December 12, 1987; NIH Guide for the Care and Use of Laboratory Animals, NIH Publication No. 86–23, 1985). All efforts were made to minimize the number of animals used in this study and their suffering.

### Radioactive labeling of bacterial melanin

BM was radioactively labeled by the iodobead method [24]. Briefly, 100 μl of 0.1% SB3-14 (sulphobetaine) in 0.25 M sodium phosphate buffer solution and 2 mCi ^131^I were collected into a tube to prevent the aggregation. The iodiobead added mixture was incubated for 5 min at room temperature. BM (10 μg) was added and the mixture was incubated for 5 min and then added to a previously-washed gel filtration medium (G-10 Sephadex) column and eluted with 0.1% SB3-14 in 0.25 M sodium phosphate buffer, pH 7.4. I-BM eluted in fraction 8 (retention factor - RF was 1.8%) with 85% precipitation in acidified brine (30% trichloroacetic acid in a saturated solution of NaCl).

### Measurements of I-BM uptake

Rats were anesthetized with 40% urethane (intraperitoneal, 1.2 g / kg). The right jugular vein and the left carotid artery were exposed and 0.4 ml of lactated Ringer’s solution containing 1% bovine serum albumin (LR-BSA) and 250,000 cpm I-BM was injected into the jugular vein. Six of the animals were also injected with 50 μg/rat of unlabeled BM, to determine the nature of melanin uptake. At various time points (5, 20, 30, 45 and 60 min) after the iv injection, blood was obtained from the carotid artery. In rats injected with cold BM arterial blood was sampled every 1 minute. The arterial blood was centrifuged to collect arterial serum. The levels of radioactivity were determined for the serum, brain, liver and kidney tissues in a gamma counter. Results were expressed as tissue/serum ratios (μl/g) of I-BM. After the last sample was obtained, the animals were perfused systemically and then decapitated (see below).

**Figure 1:**
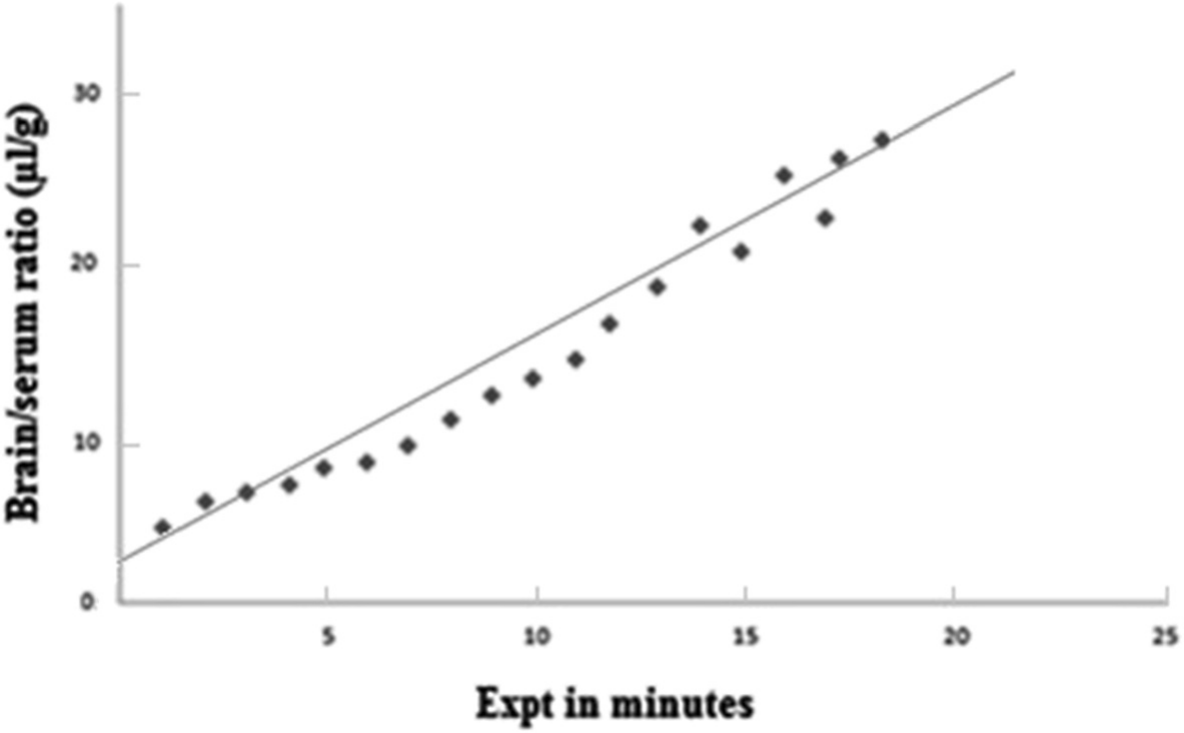
**Graph of influx of bacterial melanin into brain (A**_**br**_**/(Ĉp · t)) against exposure time ([∫Cp(T)dT]/cpt).** The solid line represents the linear fit of all the data points. The slope of the line is equivalent to K_I_ (rate of unidirectional solute flux from plasma across the BBB) and the ordinate intercept is equivalent to V_I_ (functional distribution volume of the substance that exchanges with plasma).

The unidirectional influx rate (K_i_, in units of μl/g-min) into brain was derived by multiple-time regression analysis [25] using the formula:

$$K_{r}=A_{\mathrm{br}}/\left(\hat{C}\mathrm{pt}\right)\text{,}$$

where A_br_ is the amount in the extravascular compartments of brain per unit mass of brain tissue at time t, Ĉp is the amount of radioactivity in serum at time t. The brain/serum ratio was plotted against exposure time (Figure 1). Exposure time (Expt) was calculated as:

$$\mathrm{Expt}=\left[\int \mathrm{Cp}\left(T\right)\mathrm{dT}\right]/\mathrm{cpt}$$

[26] where Cp(T) is the concentration in arterial serum, and T is the dummy variable for time, dT is the apparent diffusion constant through brain tissue at 37°C.

When a test substance moves unidirectionally from plasma into brain tissue the following relationship exists [26]:

$$A_{\mathrm{br}}/\left(\hat{C}p\cdot t\right)=K_{I}\left[\int \mathrm{Cp}\left(T\right)\mathrm{dT}\right]/\mathrm{cpt}+V_{I}$$

where K_I_ is the rate of unidirectional solute flux from plasma across the barrier into the brain divided by the plasma concentration, and V_I_ is the functional distribution volume of the substance that exchanges with plasma.

Arterial serum obtained at different time points after the iv injection of I-MB was allowed to clot and 50 μl of the resulting serum added to 250 μl of LR-BSA and then to 250 μl of acidified brine containing 30% trichloroacetic acid. The mixture was stirred and centrifuged at 5600 × g for 15 min at 4°C. The supernatant and precipitate were separated and counted. The results were expressed as the percent of total precipitated amount.

### Brain perfusion procedure

**Figure 2:**
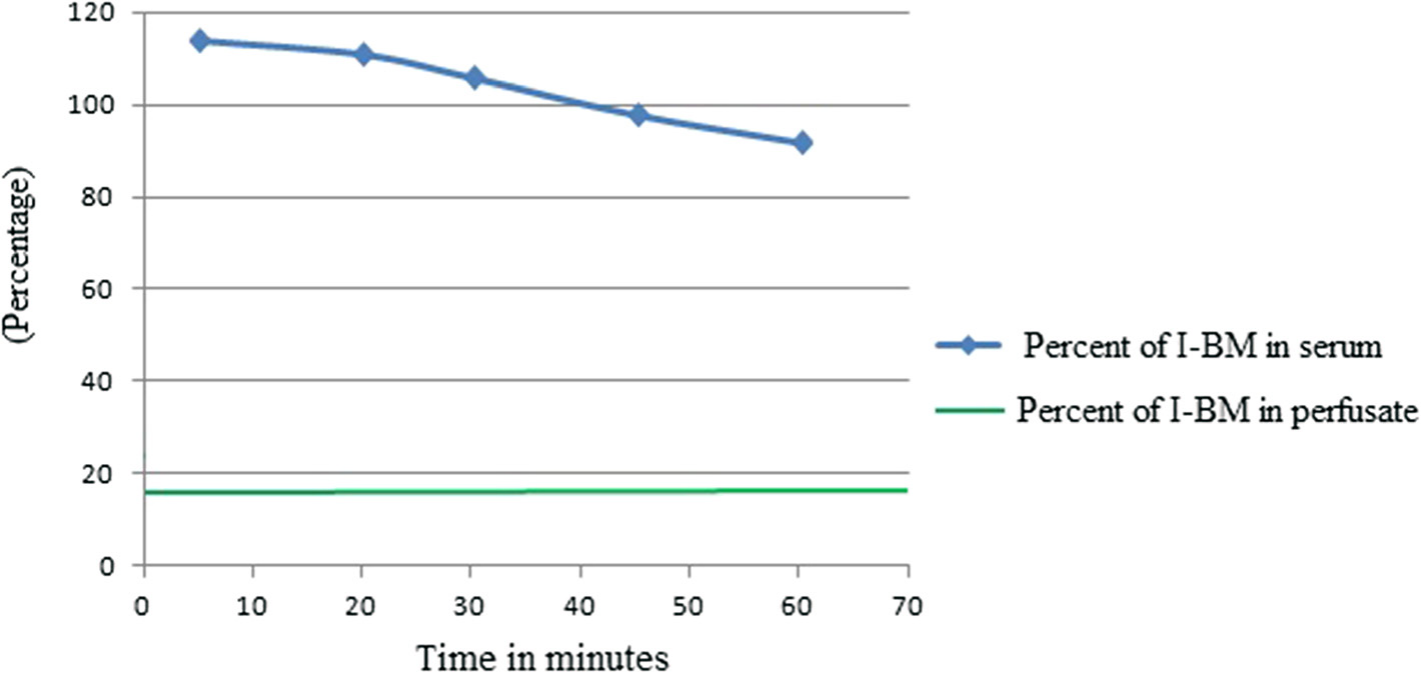
**Precipitation of radioactivity in serum.** Results are shown as the percent of control radioactivity that precipitated with acidified brine. The green line indicates percent of radioactivity in the perfusate. Figure shows that the radioactivity in serum declined over time.

Brain perfusion with saline was performed to remove the blood from the brain microvessels before decapitation because the radioactivity in brain microvessels could be a confounding factor in the correct interpretation of the data from brain parenchyma.Under urethane anesthesia, a 1–1.5 cm-long incision was made with the scissors midway between the trachea and esophagus and the shoulder of the rat. Subcutaneous fat and muscle tissues were removed using curved microdissecting forceps to expose the right carotis communis artery (CCA) and the bifurcation of the right external and internal carotid artery (ECA and ICA). The ECA was ligated with a 4.0 suture rostrally (towards the snout) after the bifurcation of the CCA into the right ECA and ICA. Loose ligature was placed around the CCA caudally (towards the tail) before the bifurcation of the right ECA and ICA. Small arterial branches of ICA and ECA were cauterized using electrocautery. The PE50 polyethylene perfusion cannula was filled with Krebs-bicarbonate buffer and connected to a 20 ml syringe using a cut 22-G needle. The syringe containing the saline solution was placed in the infusion pump. The CCA was catheterized caudally (before the bifurcation) with the buffer-filled perfusion cannula (with the tip of the cannula pointing towards the head) and fixed using a 4.0 suture. The chest cavity of rat was opened quickly and the cardiac ventricles severed. The pump was turned on to start the perfusion. The brain was perfused for 3 min at a rate of 5 ml/min. An aliquot (0.25 ml) of the perfusate was obtained for test compound concentration analysis (Figure 2). The procedure was performed in the shortest possible time period to prevent cerebral ischemia. After the perfusion, rats were quickly decapitated.

### Tissue preparation

The whole brain, spinal cord, liver and kidney were obtained from each rat after decapitation. The brains were dissected into 10 regions (frontal cortex, parietal cortex, occipital cortex, substantia nigra, hippocampus, hypothalamus, thalamus, pons medulla, cerebellum, and midbrain) according to the method of Glowinski & Iversen [27] and the spinal cord divided into 3 regions: cervical, thoracic, and lumbar. In their study, Glowinski & Iversen also isolated the striatum but we have included the substantia nigra as this region is the target of our research project. The substantia nigra was dissected bilaterally by a special razor. The cuts were made caudal to the mammillary bodies, rostral to the pons, lateral to the interpeduncular nucleus, and along a line extending through the medial lemniscus to the lateral edge of the brain stem. The sample included substantia nigra pars compacta and pars reticulata. Brain and spinal cord samples were weighed, the level of radioactivity measured as described below, and the results expressed as the brain/serum ratios in μl/g.

The samples were homogenized with a glass homogenizer in 3 ml of LR-BSA and then centrifuged at 5600 × g for 10 min at 4°C. An aliquot of 1.5 ml of the supernatant was added to 1.5 ml of acidified brine containing 30% trichloroacetic acid. The mixture was stirred and then centrifuged at 5600 × g for 10 min at 4°C. The supernatant and precipitate were separated and counted. The results were expressed as the percentage of total precipitated counts. The same procedure was used for the liver and kidney samples.

To estimate degradation rate during the process of acid precipitation, I-BM was added to non-radioactive arterial whole blood and to whole brain. These samples were then processed according to the above described procedure and the percentage of total precipitated counts was determined. The mean value obtained from controls was 78% for serum and 67% for brain. The values for the experimental samples were divided by the value obtained from control and multiplied by 100 to get the final results.

### Statistical processing

Mean values of measurements are presented with their standard deviations. Mean values were compared using *t*-test. One-way ANOVA was used to compare regional uptake rates to whole brain.

## Results and discussion

In Table 1 the results for brain and serum are compared. The degradation rate of I-BM was significantly lower in brain over 60 min, whereas degradation in blood was seen at 30 min but with a significant level of non-degraded I-BM still in serum at 60 min. The curve in Figure 1 shows the uptake of I-BM by brain in rats co-injected with non-labeled bacterial melanin. Brain uptake was almost linear for 20 min of exposure after i/v injection with a Ki of approximately 1.0 ul/g-min. However, there was a statistically significant decrease in the Ki for brain after 40 minutes of exposure: 0.218 ± 0.08 (*p* < 0.05, Table 2). This shows that transport of BM across the BBB involves a saturable transport system and the influx of BM into the CNS prevails over the efflux. Table 3 shows the uptake of BM into brain and spinal cord regions. Statistical comparison of the whole brain value to brain regions showed a significant variation (*p* < 0.01). The hypothalamus, thalamus, substantia nigra and lumbar region of the spinal cord showed higher uptake in comparison to whole brain. The highest uptake rate was in the thalamus and substantia nigra. In rats injected with mixture of I-BM and 50 μg/rat of cold BM, inhibition of uptake was revealed for the whole brain. Table 2 shows that inclusion of unlabeled BM significantly inhibited the efflux of I-BM. The values for whole brain were estimated by adding together the levels of radioactivity and weights for all the brain regions. To evaluate the elimination routes of I-BM, brain, liver and tissue samples were obtained. The rates of I-BM uptake by different tissues are presented in Table 2. The highest rate was registered for the substantia nigra. The uptake rate was significantly higher also in lumbar spinal cord and thalamus.

**Table 1:** Precipitation of radioactivity from brain and serum.

	**5 min**	**20 min**	**30 min**	**45 min**	**60 min**
**Brain (% of control)**	122 ± 3.6	118 ± 1.8	112 ± 1.6	107 ± 1.4	104 ± 0.90
**Serum (% of control)**	114 ± 2.4	111 ± 3.9	106 ± 4.4	98 ± 3.2	92 ± 3.4

Results are shown as the percent of control radioactivity that precipitates with acidified brine. Means ± SD, for the group (n = 6).

**Table 2:** Rates of radioactive bacterial melanin uptake by brain, liver and kidneys after intravenous bolus injection

	**Brain**	**Liver**	**Kidney**
**Uptake rate of I-BM**	0.218 ± 0.07	9.7 ± 2.4	17.4 ± 3.3
**Uptake rate of I-BM mixed with 50 μg/rat of cold BM**	0.084 ± 0.008*	4.4 ± 1.2	18.2 ± 4.1

Data are expressed as μl/g-min 40 minutes after injection. Means ± SD for the group (n = 6), *P < 0.05.

**Table 3:** Uptake of bacterial melanin by CNS and CNS regions after intravenous bolus injection

**CNS region**	**Tissue/serum ratio (μl/g)**
Frontal cortex	29.5 ± 1.9
Parietal cortex	22.2 ± 2.7
Occipital cortex	21.8 ± 1.8
Hypothalamus	36.6 ± 6.3
Thalamus	44.8 ± 8.4
Substantia nigra	60.7 ± 10.2
Hippocampus	28.2 ± 3.1
Cerebellum	32.1 ± 3.2
Midbrain	29.4 ± 2.8
Pons medulla	26.5 ± 1.8
Cervical spinal cord	33.3 ± 2.9
Thoracic spinal cord	28.2 ± 2.8
Lumbar spinal cord	58.2 ± 7.3
Whole CNS	26,7 ± 2.8

Results are expressed as tissue/serum ratios (μl/g). Means ± SD for the group (n = 6).

The purpose of the study was to test the ability of BM to cross the BBB. BM has been studied in various models of neurodegeneration and the results suggest that it reaches the brain parenchyma, as it induces significant regeneration in CNS after trauma and accelerates motor recovery [22]. In a previous series, studying effects of BM on regeneration and motor recovery after destruction of sensorimotor cortex, we have tested the availability of BM in the brain tissue by HPLC (Petrosyan TR, unpublished data), although we did not study the kinetics of BM in the whole organism. HPLC confirmed the presence of structurally-intact BM in the brain tissue after injection. In the present work we focused mostly on the pharmacokinetic aspects to obtain evidence for the ability of melanin to cross the BBB. This confirmed the ability of BM to cross BBB and reach brain tissue. Co-injection of excess amount of unlabeled BM showed that transport of radioactively labeled I-BM across the BBB is saturable. Entry of BM into the brain was linear for the first 20 exposure minutes and the influx itself was saturable, meaning that transport of BM through BBB was basically unidirectional. The influx rate in BM co-administered rats started to decrease after 20 minutes. BM showed enzymatic stability in both blood and brain tissue. This suggests that blood-introduced BM could contribute significantly to brain levels of BM. Labeled BM had a significant residence time in blood, so any amount of BM introduced into the bloodstream has a significant period to cross the BBB. Moreover, brain residence time appeared to be longer than the blood residence time. Therefore BM has a favorable pharmacokinetic profile for use as a therapy for neuroprotection. BM uptake rate differed widely throughout the CNS and the thalamus and substantia nigra had higher uptake rates than other regions. Research interest was focused on the substantia nigra as it is affected by neurodegeneration. The lumbar spinal cord showed the second highest uptake rate of any CNS region with a significant saturable component. The uptake of BM by a number of brain regions raises the possibility that it may be affecting various aspects of brain function or even altering BBB function.

Despite the fact that BM can cross the BBB, it is not known whether BM uses a melanin transporter to do so. There is not much data on the transport of melanin and its mediators. However, Berliner *et al.* mentioned the possible role of melanin as a transporter that crosses the BBB and has a potential for pharmacological application as a transporter [28]. For example, melanin easily binds to nerve growth factor, and transports it across the blood–brain barrier. By transporting nerve growth factor across the blood–brain barrier and increasing the permeability of the blood–brain barrier, melanin is useful in allowing nerve growth factor to reach the brain tissue [29]. To add more information to the pharmacokinetic profile of the BM we also tested the uptake rate of I-BM into liver and kidneys. Results showed that uptake rate was almost two-fold higher in kidneys, meaning that bacterial melanin accumulates in the kidneys and less so in the liver.

## Conclusions

In summary, BM is enzymatically stable in blood and in brain parenchyma and is transported by a saturable mechanism into the CNS parenchyma. Uptake from blood occurs throughout the CNS and is particularly high for the substantia nigra, hypothalamus, thalamus and lumbar spinal cord. Radioactively labeled BM is more stable in brain, suggesting that BM introduced into the blood or peripheral tissue (intramuscular injection) could contribute to the levels of melanin in CNS parenchyma.

## Competing interests

The authors declare not to have a conflict of interest.

## Authors’ contributions

TRP carried out labeling of bacterial melanin and brain perfusion procedure, performed analytical part of the study, and drafted the manuscript. HAS participated in the study, helped to draft the manuscript. All authors approved the final manuscript.

## References

[B1] Pardridge WM The blood–brain barrier: bottleneck in brain drug development NeuroRx 2005 2 1 3 14 15717053 10.1602/neurorx.2.1.3 PMC539316

[B2] Pardridge WM Brain drug targeting: the future of brain drug development Mol Interv 2003 3 2 90 105 14993430 10.1124/mi.3.2.90

[B3] Boje KM In vivo measurement of blood–brain barrier permeability Curr Protoc Neurosci 2001 Chapter 7 Unit7.19 10.1002/0471142301.ns0719s15 18428529

[B4] Wild AR Jones S Gibb AJ Activity‒dependent regulation of NMDA receptors in substantia nigra dopaminergic neurones J Physiol 2014 592 4 653 668 24344168 10.1113/jphysiol.2013.267310 PMC3934707

[B5] Forslin Aronsson A Spulber S Popescu LM Winblad B Post C Oprica M Schultzberg M alpha-Melanocyte-stimulating hormone is neuroprotective in rat global cerebral ischemia Neuropeptides 2006 40 65 75 16414116 10.1016/j.npep.2005.10.006

[B6] Chen G Frøkiær J Pedersen M Nielsen S Si Z Pang Q Stødkilde-Jørgensen H Reduction of ischemic stroke in rat brain by alpha melanocyte stimulating hormone Neuropeptides 2008 42 331 338 18359516 10.1016/j.npep.2008.01.004

[B7] Bharne AP Upadhya MA Kokare DM Subhedar NK Effect of alpha-melanocyte stimulating hormone on locomotor recovery following spinal cord injury in mice: Role of serotonergic system Neuropeptides 2011 45 25 31 21036396 10.1016/j.npep.2010.10.001

[B8] Proctor PH Free Radicals and Human disease CRC Handbook of Free Radicals and Antioxidants, Volume 1 1989 Florida, USA: CRC Press Inc 209 221

[B9] Sarna T Properties and function of the ocular melanin – a photobiophysical view J Photochem Photobiol 1992 12 215 258 10.1016/1011-1344(92)85027-r 1635010

[B10] Zecca L Tampellini D Gerlach M Riederer P Fariello RG Sulzer D Substantia nigra neuromelanin: structure, synthesis, and molecular behaviour J Clin Pathol Mol Pathol 2001 54 414 418 PMC1187132 11724917

[B11] Proctor PH Reynolds ES Free radicals and disease in man Physiol Chem Phys Med NMR 1984 16 175 195 6393156

[B12] Zecca L The absolute concentration of nigral neuromelanin, assayed by a new sensitive method, increases throughout the life and is dramatically decreased in Parkinson’s disease FEBS Lett 2002 16 216 220 11801257 10.1016/s0014-5793(01)03269-0

[B13] Hathaway JD Haque A Insights into the role of PAX-3 in the development of melanocytes and melanoma Open Cancer J 2014 4 1 6 24790680 10.2174/1874079001104010001 PMC4002046

[B14] Jacobson SG Cideciyan AV Aleman TS Sumaroka A Roman AJ Gardner LM Prosser HM Mishra M Bech-Hansen NT Herrera W Schwartz SB Liu XZ Kimberling WJ Steel KP Williams DS Usher syndromes due to MYO7A, PCDH15, USH2A or GPR98 mutations share retinal disease mechanism Hum Molec Genet 2008 17 15 2405 2415 18463160 10.1093/hmg/ddn140 PMC2733815

[B15] Mann DM Yates PO Marcyniuk B Changes in nerve cells of the nucleus basalis of Meynert in Alzheimer’s disease and their relationship to ageing and to the accumulation of lipofuscin pigment Mech Ageing Dev 1984 25 189 204 6202988 10.1016/0047-6374(84)90140-4

[B16] Aghajanyan AE Hambardzumyan AA Hovsepyan AS Asaturian RA Vardanyan AA Saghiyan AA Isolation, purification and physicochemical characterization of water-soluble Bacillus thuringiensis melanin Pigment Cell Res 2005 18 130 135 15760342 10.1111/j.1600-0749.2005.00211.x

[B17] Popov YG Effect of Melanin Culture Liquid of Bacillus Thuringiensis on the Growth and Development of Plants in an Isolated Culture Proceedings of II Moscow International Congress of Biotechnology: A Status and Prospects of Development, Part I 2003 Moscow: Nova Science Publishers, Inc 222 223

[B18] Azaryan KG Petrosyan MT Popov YG Martirosyan GS Melanin in the agriculture and dendrology Bull Armenian Agric Acad 2004 4 7 10

[B19] Sarkissian JS Galoyan AA Kamalyan RG Chavushyan VA Meliksetyan IB Poghosyan MV Gevorkyan OV Hovsepyan AS Avakyan ZE Kazaryan SA Manucharyan MK The effect of bacterial melanin on electrical activity of neurons of the substantia nigra under conditions of GABA generation Neurochem J 2007 1 227 234

[B20] Gevorkyan OV Meliksetyan IB Ovsepyan AS Sagiyan AS Effects of BT-melanin on recovery of operant conditioned reflexes in rats after ablation of the sensorimotor cortex Neurosci Behav Physiol 2007 37 5 471 476 17505797 10.1007/s11055-007-0037-0

[B21] Gevorkyan OV Meliksetyan IB Petrosyan TR Hovsepyan AS Agadzhanyan AE Saghiyan AS Study of the influence of bacterial melanin on brain plasticity Neurochem J 2008 2 4 308 309

[B22] Petrosyan TR Gevorkyan OV Meliksetyan IB Hovsepyan AS Manvelyan LR Neuroprotective action of bacterial melanin in rats after pyramidal tract lesions J Pathophysiol 2012 19 71 80 10.1016/j.pathophys.2011.12.003 22366100

[B23] Petrosyan TR Chavushyan VA Hovsepyan AS Bacterial melanin increases electrical activity of neurons in Substantia Nigra pars compacta J Neur Transm 2014 121 259 265 10.1007/s00702-013-1095-9 25006618

[B24] Blasberg RG Fenstermacher JD Patlak CS Transport of α-aminoisobutyric acid across brain capillary and cellular membranes J Cereb Blood Flow Metab 1983 3 8 32 6822623 10.1038/jcbfm.1983.2

[B25] Patlak CS Blasberg RG Fenstermacher JD Graphical evaluation of blood-to brain transfer constants from multiple-time uptake data J Cereb Blood Flow Metab 1983 3 1 7 6822610 10.1038/jcbfm.1983.1

[B26] Blasberg RG Patlak C Fenstermacher JD Intrathecal chemotherapy brain tissue profiles after ventriculocisternal perfusion J Pharmacol Exp Ther 1975 195 73 83 810575

[B27] Glowinski J Iversen LL Regional studies of catecholamines in the rat brain. I. The disposition of [3H]norepinephrine, [3H]dopamine and [3H]dopa in various regions of the brain J Neurochem 1966 13 655 669 5950056 10.1111/j.1471-4159.1966.tb09873.x

[B28] Berliner DL Erwin RL McGee DM Methods of Treating Parkinson’s Disease using Melanin 1993 United States: Patent 5210076 A

[B29] Levi-Montalcini R The nerve growth factor: thirty-five years later EMBO J 1987 6 5 1145 1154 3301324 10.1002/j.1460-2075.1987.tb02347.x PMC553912

